# Environmental Risk Perception and Preventive Behavior during the COVID-19 Pandemic in Central Taiwan

**DOI:** 10.3390/ijerph18189920

**Published:** 2021-09-21

**Authors:** Kuo-Wei Hsu, Jen-Chih Chao, Ching-Yi Hsu

**Affiliations:** 1 Department of Landscape and Urban Design, Chaoyang University of Technology, Taichung City 413, Taiwan; kwhsu@cyut.edu.tw; 2 Independent Researcher, Taichung City 413, Taiwan; g9929704@yuntech.org.tw

**Keywords:** environmental risk perception, preventive behaviors, COVID-19, risk governance

## Abstract

Due to traffic and industrial and seasonal air pollution, wearing masks outside the home has long been a daily habit for many people in Taiwan. After the emergence of the novel coronavirus (COVID-19), which has an incubation period of up to 14 days, wearing masks and maintaining social distancing was advised to reduce exposure to this new environmental risk. This study investigates open and semi-open spaces in three districts in central Taiwan, using a non-participant observation method, with the aim of understanding people’s mask-wearing behavior. The results indicate that mask-wearing rates were higher in urban areas than in rural ones and among females than males. By age cohort, mask-wearing was most prevalent among young adults and middle-aged people and least prevalent among minors, with the elderly occupying a middle position. Masks were also more likely to be worn in semi-open spaces than in open ones. This study enriches our understanding of environmental risk perception of the pandemic and of public perceptions, which are vital to increasing the adoption of preventative measures.

## 1. Introduction

On 21 January 2020, the first confirmed case of COVID-19 appeared in Taiwan (TCDC, 2021) [1]. According to a World Health Organization (WHO) announcement from the same month, common symptoms of the new coronavirus included fever, cough, fatigue, sputum production, smell and taste abnormalities, and diarrhea, and the incubation period—i.e., the time that elapsed between infection and presentation of symptoms—was up to two weeks (WHO, 2020) [2]. This prompted governments in East Asia to impose restrictions, announce social-distancing measures, ban large-scale gatherings, and restrict other activities. The Taiwan Centers for Disease Control (TCDC) expressed an opinion that wearing masks would reduce the risk of infection (TCDC, 2021) [1]. As a result, it listed masks as anti-epidemic materials and allowed the public to purchase nine masks every two weeks.

At the time of writing, the number of deaths and infections continues to increase. As of 30 June 2021, the number of confirmed cases worldwide was 181,764,498. The United States had the highest number of infections, followed by India, Brazil, France, Russia, Turkey, and the United Kingdom (NCHC, 2021) [3]. Because the virus is transmitted via nasal and oral secretions, direct or indirect contact with such secretions, or prolonged exposure to a confirmed patient in a confined space within 2 m without respiratory protection, increases the likelihood of human-to-human infection (TCDC, 2021) [1]. Thus, the characteristics of space and the distance between people are factors that increase the infection rate, and scholars have confirmed governmental suggestions that wearing a mask can reduce the risk of infection (Cook, 2020; Greenhalgh et al., 2020; Howard et al., 2021) [4,5,6].

Mask-related research has focused on topics including masks’ comfort, design, materials, and tightness (Tsai, 2015; Luo, 2017; Hung, 2018; Li, 2019) [7,8,9,10]. However, published research on preventive behaviors has pointed out that wearing a mask may be mistaken for a sign of illness or become a target of discrimination (Feng et al., 2020; Howard et al., 2021) [4,11]. Studies of environmental risk perception, meanwhile, have mainly discussed public facilities and neighboring facilities (Wu, 2014; Chao, 2019) [12,13], and rarely discussed the behavior of people wearing masks in different environmental spaces or for different activities. This paper, therefore, seeks to understand how spatial differences, environmental risk perceptions, and the transmission characteristics of COVID-19 affect whether people proactively wear masks for self-protection against COVID-19 when entering open and semi-open spaces.

Two non-participant observations were conducted, one month after becoming COVID-19-free and eight months after the new local case of COVID-19 had been reported in Taiwan, but when infection numbers worldwide were still increasing, and vaccines were not yet being administered. The authors’ specific aims were (1) to observe the environmental risk perceptions and preventive behaviors of people in two general types of spatial environments, open (parks) and semi-open ones (markets); (2) to investigate whether and how people’s risk perceptions and preventive behaviors were affected by their background characteristics, especially gender and age; and (3) to investigate the relationship between people’s mask-wearing behaviors and their risk perceptions.

Given the severity of the pandemic and its rapid progress, Taiwan sought to control the spread of local cases from the end of 2019 to May 2021. Mask-wearing behavior was encouraged both for self-protection and the protection of others from infection. Conversely, not wearing masks was deemed to elevate environmental risk, increase the numbers of infection clusters, disrupt daily-life routines, and even boost the risk that lockdowns would have to be implemented to reduce the spread of the virus. The results are expected to have important implications for those tasked with making and implementing public-health policy by providing them with a clearer understanding of who wears masks, when, where, and why.

## 2. Literature Review

While the COVID-19 pandemic raged around the world, Taiwan was not affected much before a local outbreak on 15 May, 2021. Nevertheless, the media and information environment stimulated Taiwanese people to become more aware of environmental disease risks and to reduce their risk of infection through taking personal precautions. TCDC continues to advocate social distancing of 1.5 m indoors and 1 m outdoors.

### 2.1. Environmental Risk Perception

People are exposed to different risks and hold varying perceptions of those risks across different environments. When people approach or enter a place where they think they are at risk, they conduct their own informal risk assessments based on its characteristics to reduce their exposure. However, Flin et al. (1996) pointed out that humans do not rely on a rational, scientific approach when assessing the risks they may encounter on a daily basis but rather adopt a subjective quantitative assessment and engage in various activities based on its perceived results [14]. Various other studies have likewise used the term risk perception to refer to these subjective evaluations of and attitudes toward risk (Bauer, 1960; Cutter, 1993; J. Wang, 2000; T. Wang, 2004) [15,16,17,18]. The environments being evaluated as higher-risk may include their own living spaces, if these are—for example—next to gas stations (Wu, 2014) [12] or in industrial areas that might produce contaminants (Janmaimool and Watanabe, 2014; Dettori et al., 2020) [19,20].

Risk perception is in part determined by uncertainty and fear (Slovic, 2000) [21]. Individuals’ risk perceptions of the pandemic vary significantly due to their wide range of perceptions of life, death, uncertainty (Shen et al., 2020) [22], and the severity of the event itself (Shahin and Hussien, 2020) [23]. Due to the transmission characteristics of COVID-19, the risk of infection is higher in confined, poorly ventilated, and/or crowded spaces. Close contact with other people and, possibly, touching surfaces increases the environmental risk of infection with this virus, and knowledge of these factors can reasonably be expected to affect people’s environmental risk perceptions and preventive behaviors in different spaces. The risk of infection is higher in environments where people may come into close contact with each other, and wearing a mask can reduce that environmental risk due to the characteristics of the virus (TCDC, 2021) [1]. In a survey conducted among Taiwanese people by Tsai et al. (2021) in October 2020, 63.5% of respondents reported feeling that the COVID-19 epidemic in Taiwan was not serious [24]. Kuang et al. (2020) investigated COVID-19 awareness, risk perception, and stress in India at the end of May 2020, and the vast majority reported perceiving that their risk of personally contracting coronavirus was low (23%) to nonexistent (60%) [25].

### 2.2. Preventive Behavior

People’s perceptions of the severity of the COVID-19 pandemic are related to their awareness of risks and are critical factors in encouraging them to participate in disease-prevention measures (Shahin and Hussien, 2020) [23]. In general, individuals who perceive that they have a high level of susceptibility to a particular disease will adopt necessary measures to reduce the risk of developing it (Janz, 1984) [26], and confronted by a major disease outbreak, most people feel at risk, and therefore, take steps to protect themselves (Berni et al., 2021) [27], such as washing hands, wearing masks, avoiding eating out, getting vaccinated, reducing contact with others, maintaining social distancing, disinfecting surfaces and their hands with alcohol, taking their temperature regularly, and staying at home as much as possible. Several studies have concluded that COVID-19 preventive measures, such as hand hygiene, social distancing, wearing face masks, and avoiding crowded areas, are essential to controlling the spread of this virus (Akalu et al., 2020; Ojo et al., 2020; Okoro et al., 2020; Nwagbara et al., 2021) [28,29,30,31]. According to TCDC, however, wearing a mask is the single most effective measure both for enhancing one’s self-protection from COVID-19 and for protecting others; and this is even more true in confined spaces with moderate to poor ventilation (TCDC, 2021) [1]. Nevertheless, few studies have hitherto compared COVID-19 prevention behaviors across open and semi-open spaces.

## 3. Methods

Non-participant observation is a type of observational research that is unlike its better-known counterpart, participant observation, in which the observed persons are aware of the presence of the observer, and therefore the behavior exhibited by all parties may differ from their usual behavior. Non-participant observation research, in contrast, reduces this impact of subjects knowing that they are being observed (e.g., Chang and Tu, 2013; Wang et al., 2014) [32,33]. By directly observing the behavior of individuals and groups, researchers can simultaneously record and analyze shifts in social reality during the pandemic, without people changing their behavior as a result of knowing that research is being conducted. Therefore, this study adopted it. Data collection involved making on-site observation records and photographs and focused on whether the behavior of mask-wearers varied when entering different spaces and whether differences in terms of age or gender could be observed.

The study was undertaken to observe mask-wearing behavior according to genders (male, female) and age groups (0–17, 18–64, and over 65 years old). Three districts were chosen as case-study sites, providing varied population sizes/densities. They were the Nantun District of Taichung (population 176,844), the East District of Taichung (population 75,538), and Xiushui Township, Changhua County (population 38,872) (Civil Affairs Bureau; Department of Civil Affairs, 2021) [34,35].

**Figure 1:**
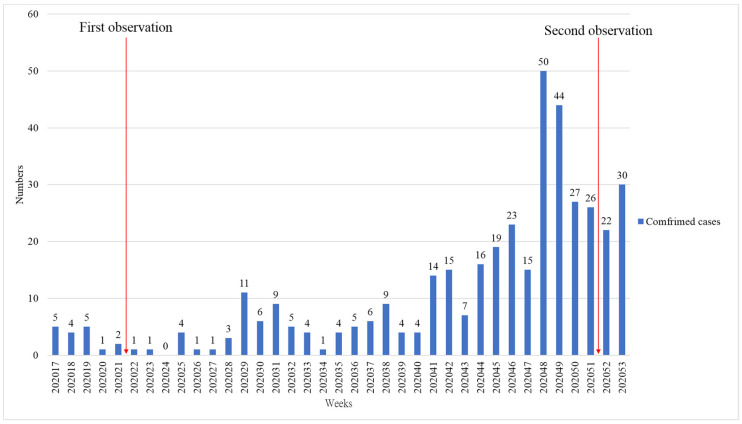
Confirmed COVID-19 cases in Taiwan, by date (TCDC, 2021) [1].

The first observation, on 17–18 May, 2020, was conducted during a period when Taiwan had no local COVID-19 cases, and its government did not have any regulations about mask-wearing in place. However, on 1 December, 2020, with the pandemic still developing rapidly, Taiwan’s government stepped up its promotion of mask-wearing, and began to positively require it in certain locations. As shown in Figure 1, the second of the present study’s two observation windows was after that change, i.e., on 28–29 December, 2020. Average monthly temperatures for May and December that year were 27.5 and 19.5 °C, respectively (Central Weather Bureau, 2021) [36]. Each site was observed for one hour, and photographs and video recordings were taken (see Table 1) alongside the observers’ notes.

The three open spaces observed were Wenxin Park, Lecheng Park, and Xiushui Park, and the five semi-open ones, Dalong Twilight Market, Wenxin Twilight Market, Shinkong Twilight Market, Jianguo Market, and Xiushui Twilight Market. Wenxin Park, Dalong Twilight Market, and Wenxin Twilight Market are all in Nantun. Lecheng Park, Shinkong Twilight Market, and Jianguo Market are in the East District; and Xiushui Park and Xiushui Twilight Market are in Xiushui.

## 4. Results

In the first observation, a total of 2325 people were observed in the eight selected locations in the three focal districts, and their overall mask-wearing rate was 42%. In Nantun District’s Wenxin Park, the number of people observed on that occasion was 162, of whom 33% were wearing masks. No elderly men did so, and the rate for non-elderly adult males was 16%, as compared to 38% for non-elderly adult females. At Nantun’s Dalong Twilight Market, 330 subjects were observed during the May observation window, and their overall mask-wearing rate was 40%. Among elderly and non-elderly adult women, the rates were 57% and 59%, respectively. In Nantun’s Wenxin Twilight Market in May, there were 204 subjects, whose overall mask-wearing rate was 56%, but much higher among females, i.e., 83%, 66%, and 63% for female minors, non-elderly women, and elderly women, respectively. The combined average mask-wearing rate for the three observation sites in Nantun District in May was 43%.

Turning now to the May observations in the East District of Taichung City, the number of observations in Lecheng Park was 201, and the overall mask-wearing rate was the lowest observed anywhere in May, 16%, with that of minors (6%) and elderly men (9%) both being markedly lower than the site average. The number of people observed in the Shinkong Twilight Market was 471. Their overall mask-wearing rate was 57%, but considerably higher among non-elderly women (72%). The overall mask-wearing rate in the third East District observation site, Jianguo Market, was 61%, based on 393 people observed, but much higher for non-elderly and elderly women, at 72% and 81%, respectively. All other age and gender categories at the same site on the same occasion had mask-wearing rates above 50%, and the combined average mask-wearing rate for the East District was 45%.

At the final sites, Xiushui Park and Xiushui Twilight Market in Changhua, the overall mask-wearing rates were 26% and 43%, respectively. In Xiushui Park, where 81 people were observed, the average mask-wearing rates of minors and seniors was less than 16%. The number of people observed in Xiushui Twilight Market was 483, and their overall mask-wearing rate was 43%, but this was much higher—65%—among elderly females. Table 2 sets forth all the mask-wearing rates from the first observation window by demographic group.

The second observation, which was conducted in the same three districts and eight locations, yielded a total of 1809 observations and an overall mask-wearing rate of 58%. In Nantun, the second observation in Wenxin Park collected data on 245 people, of whom 27% were wearing masks, but among minors, the proportion was around half the overall average. In Dalong Twilight Market, the 281 people observed had an overall mask-wearing rate of 67%, with the minors’ rate being the same as the overall rate, non-elderly adults being above the overall rate (78%), and seniors being lower (55%). The number of people observed in the Wenxin Twilight Market was 269, and their overall mask-wearing rate was 66%, but female minors were only half that likely to wear masks, whereas all other demographic groups’ rates were above 60%. The combined average mask-wearing rate for Nantun District was 53%.

During the second observation window in the East District, the number of people observed in Lecheng Park was 172, and the overall rate of mask-wearing was lower, at 20%. Only two minors were seen with masks. The non-elderly adults’ rate was close to the site average, at 21%. Of the 281 people observed in the Shinkong Twilight Market, on the other hand, 86% were wearing masks, and was somewhat higher among non-elderly adult females. In the Jianguo market on this occasion, 268 people were observed, and their overall mask-wearing rate was 70%, but among non-elderly women, it was 90%, the same as at Shinkong. The combined average mask-wearing rate for the three observation locations in East District was 59%.

Finally, in Xiushui Park, the number of people observed during the second observation window was 10, of whom four were wearing masks. In the Xiushui Twilight Market, there were 283 people, whose overall mask-wearing rate was 58%, though this rose to 84% among non-elderly adult females. The combined average mask-wearing rate for Xiushui Township during the second observation window was 66%. Full details of the second observation are presented in Table 3.

## 5. Discussion

During the first observation, the total number of people observed in open spaces across all three districts was 444, and the rate of wearing masks in those spaces was 25%. The number of people observed in the semi-open spaces on the same occasion was 1881, and their rate of mask-wearing was 51%. The relatively low rates in the former type of location—i.e., 33% in Wenxin Park, 16% in Lecheng Park, and 26% in Xiushui Park—is probably attributable to social distancing being relatively easy there, but mask-wearing being rendered difficult in some cases by people’s chosen activities, e.g., jogging. During the same observation window, in open spaces across all districts, the average rate of mask-wearing by minors was 27%; by non-elderly adults, 33%; and by the elderly, 15%. However, mask-wearing behavior varied even more sharply depending on which spatial environment they were in and precisely what they were doing, e.g., was low when using children’s playground equipment or running, but high when chatting or walking. Secondly, it should be noted that the mask-wearing rate in semi-open spaces was considerably higher than in open ones: i.e., 40% in Dalong Twilight Market, 56% in Wenxin Twilight Market, 57% in Shinkong Twilight Market, 61% in Jianguo Market, and 43% in Xiushui Twilight Market; and by age group, the rates across all of these sites were 47% or above. This probably reflects that, as of May 2020, the Taiwanese public had a higher awareness of the environmental risk of COVID-19 in places where maintaining social distancing was made more difficult by the spatial arrangements and/or the presence of large numbers of other people.

During this study’s second observation, the number of people observed in open spaces across all three districts was 427, and the rate of mask-wearing, 35%, was still low (i.e., 44% in Wenxin Park, 20% in Lecheng Park, and 40% in Xiushui Park), but markedly higher than it had been in May of the same year. On the same occasion, the total number of people observed in semi-open spaces was 1382, and their rate of mask-wearing was 69%—likewise an increase of more than a third since May. These observed increases in the prevalence of mask use may have followed the development of the epidemic; i.e., when there COVID-19 cases in Taiwan, people’s risk perceptions were heightened, even in open spaces and in rural areas, as compared to when the pandemic had started, but the country was still COVID-19-free. In semi-open spaces, mask-use rates remained not only high relative to their open-space counterparts, but on average, over a third higher than they had been during the May observation: i.e., 67% in Dalong Twilight Market, 66% in Wenxin Twilight Market, 86% in Shinkong Twilight Market, 70% in Jianguo Market, and 58% in Xiushui Twilight Market. Similarly, the rates of mask-wearing by age group were all 63% or above, as compared to around half in May. Due to announcements of COVID-19-related government policies and news reports about a new confirmed case in Taiwan, people were presumably more aware in December than in May of the risks that attended visiting congested markets and thus took more steps to defend themselves when doing so. The sharply reduced number of people observed in the markets during the second observation window—i.e., 1382 vs. 1881—could also reflect that people chose to reduce their environmental risk by avoiding crowded or confined spaces.

Based on the results of our first and second observations, the rates of mask-wearing in open and semi-open spaces rose from 22% and 54%, respectively, in mid-May 2020, when there had been no local cases in Taiwan for 30 days, to 36% and 63%, respectively, in late December, following a local confirmed case. In other words, people’s perception of environmental risks changed alongside changing data on Taiwan’s epidemic. In this study, three locations in central Taiwan with different population densities were selected to investigate whether variation in such densities, socio-economic development, and regional characteristics influenced people’s environmental risk perceptions and/or their mask-wearing as a COVID-19 countermeasure. In both observations, the rate of wearing masks in open spaces was highest in Nantun, followed by Changhua, and the lowest in East District; whereas in semi-open spaces, the mask-wearing rate was highest in the East District, followed by Nantun, and lowest in Xiushui Township in Changhua. As such, the results of this study do not suggest that the relationship between environmental risk perception and mask-wearing behavior is affected by local population density or income. That is, the mask-wearing rate was low in open spaces in both urban and rural areas. Across the two observations, meanwhile, the overall rate of mask-wearing was higher in urban than rural areas, echoing findings by Chen and Chen (2020) [37]. Our finding that women’s mask-wearing rates were higher than men’s was also consistent with prior findings by Brouard et al. (2020) [38], Galasso et al. (2020) [39], Kwok et al. (2020) [40], Raude et al. (2020) [41], Barile et al. (2021) [42], and Bronfman et al. (2021) [43]. However, it differed from the findings reported by Arslanca et al. (2021) [44]. Our direct observations suggest that this discrepancy may be due to predominately male public behaviors, such as smoking and eating betel nut. The rate of mask-wearing was higher in semi-open than in open spaces; and rates of mask-wearing by age group were highest among non-elderly adults, followed by the elderly, and were lowest among minors. The possible explanations for such results could include low awareness and inadequate knowledge of the transmission characteristics of the new disease among the elderly. Daoust (2020) [45] found that elderly people were no more disciplined, in terms of their compliance with preventive measures than other age groups—especially when it came to wearing face masks outside their homes. Minors’ mask-wearing behavior appeared to have been influenced by their parents and peers; i.e., when parents were not wearing masks, their minor children were not wearing them, either.

## 6. Conclusions

When COVID-19 broke out in Taiwan, public-health messages began to promote the wearing of masks, disinfection, and frequent hand washing; and a national policy of increasing mask production was announced, with the stated aim of enabling citizens to buy a fixed number of masks per week to reduce their odds of being infected with the virus. Although there had been no local cases in Taiwan for more than a month as of that time, foreign epidemics remained severe, and the TCDC continued to advise people to be wary of the disease and take suitable precautions. Unsurprisingly, therefore, during our first observation in May 2020, people’s mask-use behavior appeared to reflect a perception that environmental risk was higher in semi-open spaces than in open ones. By the time of our second observation, in December 2020, the rates of mask-wearing in both open and semi-open spaces were considerably higher, presumably indicating that people’s awareness of environmental risks had changed as a result of the return of COVID-19 to Taiwan. At the time of writing, wearing masks to reduce the risk of infection is still a daily habit in Taiwan and appears to have helped to suppress the occurrence of community clusters of infection there. Thus, we are able to conclude that people of both genders did change their behavior due to environmental risks and improved their self-protection measures, whether in urban or rural areas or in spaces with different characteristics. Government policy advocacy, mask supply, and public cooperation are all essential elements in maintaining effective epidemic control. This study has provided useful insights into COVID-19-related environmental risk perceptions and preventive behaviors. The chief drawback of our method was the potential lack of validity because we were unable to ask why people were acting in the ways that they did—in part because of maintaining social distancing ourselves and in part because revealing our presence would have gone against the non-participant observation protocol. Future studies should, therefore, make use of questionnaires or interviews, where allowed, to enable a deeper understanding of how environmental risk perceptions influence preventive behaviors and of the factors (temperature, culture, religion, etc.) that influence people’s choice to wear or not wear masks in various situations. Such research will benefit health professionals’ and officials’ efforts to promote appropriate disease-prevention behaviors during the remainder of the pandemic and any future similar events.

## Figures and Tables

**Table 1:** Photos of observation sites.

Location Spatial Environment	Nantun District, Taichung	East District, Taichung	Xiushui Township, Changhua
Open spaces	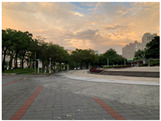	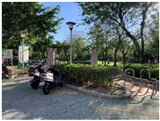	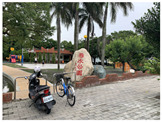
	Wenxin Park	Lecheng Park	Xiushui Park
Semi-open spaces	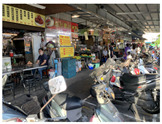	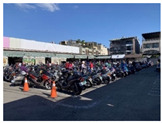	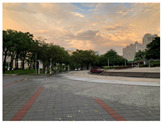
	Dalong Twilight Market	Shinkong Twilight Market	Xiushui Twilight Market
	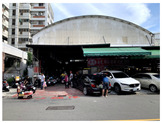	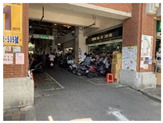	
	Wenxin Twilight Market	Jianguo Market	

**Table 2:** Rates of mask-wearing, first observation, by estimated demographic group.

Location Group (Mask-Wearing)	Wenxin Park	Dalong Twilight Market	Wenxin Twilight Market	Lecheng Park	Shinkong Twilight Market	Jianguo Market	Xiushui Park	Xiushui Twilight Market
Age 0–17, male (yes)	9	2	1	1	11	8	1	9
Age 0–17, male (no)	11	8	3	28	9	6	10	13
Mask-wearing ratio	45%	20%	25%	3%	55%	57%	9%	41%
Age 0–17, female (yes)	4	4	5	2	16	3	1	12
Age 0–17, female (no)	1	8	1	22	10	3	6	16
Mask–wearing ratio	80%	33%	83%	8%	62%	50%	14%	43%
Age 18–64, male (yes)	9	30	16	8	60	51	12	43
Age 18–64, male (no)	46	45	17	32	63	48	16	69
Mask-wearing ratio	16%	40%	48%	20%	49%	52%	43%	38%
Age 18–64, female (yes)	15	88	73	12	144	118	5	39
Age 18–64, female (no)	24	60	38	30	55	47	5	61
Mask-wearing ratio	38%	59%	66%	29%	72%	72%	50%	39%
Age over 65, male (yes)	0	13	11	3	18	25	3	30
Age over 65, male (no)	19	30	12	32	28	22	18	57
Mask-wearing ratio	0%	30%	48%	9%	39%	53%	14%	34%
Age over 65, female (yes)	4	24	17	8	38	50	1	52
Age over 65, female (no)	20	18	10	23	19	12	3	28
Mask-wearing ratio	17%	57%	63%	26%	67%	81%	25%	65%
Subtotal	162	330	204	201	471	393	81	483
Overall mask-wearing rate	33%	40%	56%	16%	57%	61%	26%	43%

**Table 3:** Rates of mask-wearing, second observation, by estimated demographic group.

Location Group (Mask-Wearing)	Wenxin Park	Dalong Twilight Market	Wenxin Twilight Market	Lecheng Park	Shinkong Twilight Market	Jianguo Market	Xiushui Park	Xiushui Twilight Market
Age 0–17, male (yes)	1	5	2	0	1	1	0	8
Age 0–17, male (no)	6	2	0	17	0	0	1	7
Mask-wearing ratio	14%	71%	100%	0%	100%	100%	0%	53%
Age 0–17, female (yes)	1	5	2	2	8	1	0	15
Age 0–17, female (no)	7	3	4	20	3	2	0	7
Mask-wearing ratio	13%	63%	33%	9%	73%	33%	0%	68%
Age 18–64, male (yes)	35	41	33	4	47	43	3	43
Age 18–64, male (no)	74	18	22	16	20	31	5	27
Mask-wearing ratio	32%	69%	60%	20%	70%	58%	38%	61%
Age 18–64, female (yes)	60	142	128	6	162	99	1	114
Age 18–64, female (no)	50	22	39	21	18	11	0	21
Mask-wearing ratio	55%	87%	77%	22%	90%	90%	100%	84%
Age over 65, male (yes)	4	10	8	8	7	30	0	15
Age over 65, male (no)	4	14	5	38	1	13	0	11
Mask-wearing ratio	50%	42%	62%	17%	88%	70%	0%	58%
Age over 65, female (yes)	3	13	17	20	13	25	0	3
Age over 65, female (no)	0	6	9	20	1	12	0	12
Mask-wearing ratio	100%	68%	65%	50%	93%	68%	0	20%
Subtotal	245	281	269	172	281	268	10	283
Overall mask-wearing rate	44%	67%	66%	20%	86%	70%	40%	58%

## Data Availability

The data presented in this study are available in the article.
